# Assessment of COVID-19 progression on day 5 from symptoms onset

**DOI:** 10.1186/s12879-021-06596-5

**Published:** 2021-08-28

**Authors:** Elisa Gentilotti, Alessia Savoldi, Monica Compri, Anna Górska, Pasquale De Nardo, Alessandro Visentin, Giorgia Be, Elisa Razzaboni, Nicola Soriolo, Dario Meneghin, Domenico Girelli, Claudio Micheletto, Sara Mehrabi, Elda Righi, Evelina Tacconelli

**Affiliations:** 1 Division of Infectious Diseases, Department of Diagnostics and Public Health, University of Verona, P.le L.A. Scuro 10, 37134 Verona, Italy; 2 Department of Medicine, Section of Internal Medicine, University of Verona, EuroBloodNet Referral Center for Iron Metabolism Disorders, Azienda Ospedaliera Universitaria Integrata Verona, 37138 Verona, Italy; 3 Cardio-Thoracic Department, Respiratory Unit, Azienda Ospedaliera Universitaria Integrata Verona, 37124 Verona, Italy; 4 Department of Radiology, University of Verona, Piazzale L.A. Scuro, 10, 37100 Verona, Italy

**Keywords:** COVID-19, Disease progression, Outcome, Public health

## Abstract

**Background:**

A major limitation of current predictive prognostic models in patients with COVID-19 is the heterogeneity of population in terms of disease stage and duration. This study aims at identifying a panel of clinical and laboratory parameters that at day-5 of symptoms onset could predict disease progression in hospitalized patients with COVID-19.

**Methods:**

Prospective cohort study on hospitalized adult patients with COVID-19. Patient-level epidemiological, clinical, and laboratory data were collected at fixed time-points: day 5, 10, and 15 from symptoms onset. COVID-19 progression was defined as in-hospital death and/or transfer to ICU and/or respiratory failure (PaO_2_/FiO_2_ ratio < 200) within day-11 of symptoms onset. Multivariate regression was performed to identify predictors of COVID-19 progression. A model assessed at day-5 of symptoms onset including male sex, age > 65 years, dyspnoea, cardiovascular disease, and at least three abnormal laboratory parameters among CRP (> 80 U/L), ALT (> 40 U/L), NLR (> 4.5), LDH (> 250 U/L), and CK (> 80 U/L) was proposed. Discrimination power was assessed by computing area under the receiver operating characteristic (AUC) values.

**Results:**

A total of 235 patients with COVID-19 were prospectively included in a 3-month period. The majority of patients were male (148, 63%) and the mean age was 71 (SD 15.9). One hundred and ninety patients (81%) suffered from at least one underlying illness, most frequently cardiovascular disease (47%), neurological/psychiatric disorders (35%), and diabetes (21%). Among them 88 (37%) experienced COVID-19 progression. The proposed model showed an AUC of 0.73 (95% CI 0.66–0.81) for predicting disease progression by day-11.

**Conclusion:**

An easy-to-use panel of laboratory/clinical parameters computed at day-5 of symptoms onset predicts, with fair discrimination ability, COVID-19 progression. Assessment of these features at day-5 of symptoms onset could facilitate clinicians’ decision making. The model can also play a role as a tool to increase homogeneity of population in clinical trials on COVID-19 treatment in hospitalized patients.

## Introduction

The global Coronavirus disease 2019 (COVID-19) pandemic is challenging healthcare systems worldwide [1]. As of June 2021, the number of patients confirmed to have COVID-19 has exceeded 190 million in 191 countries, with more than 4 million deaths [2]. The critical disease, described in up to 20% of hospitalised patients [3, 4], is associated with high case fatality rate and leads to substantial increase in the demand for hospital beds and shortage of medical equipment. Up to January 2021, the hospital and intensive care unit (ICU) occupancy and the new admission due to COVID-19 increased in several European countries, reaching 82% of the peak ICU occupancy observed during the pandemic [5]. Descriptive studies on the natural course of COVID-19 have revealed that the disease progression occurs typically at an early stage of the illness, usually within 7–14 days after the onset of symptoms [6, 7] with or without acute severe respiratory distress syndrome [8]. Given the lack of standardized treatments for patients with COVID-19, the identification of patients at higher risk of short-term complications is of utmost importance to ensure the best possible clinical care and to optimise resource allocation. Since the beginning of the pandemic, several prognostic predictive models combining various clinical and laboratory parameters have been developed to estimate risk of patients experiencing poor outcomes. A systematic review of ten prognostic models observed that the most reported predictors of disease progression and mortality were age, sex, C-reactive protein (CRP), lactic dehydrogenase (LDH) and lymphocyte count [9].

All the published models were computed using the trend of clinical and laboratory information starting from the admission day onward [10–12], with the result that the data was related to a wide range of disease length as opposed to specific time points. To the best of our knowledge, no prognostic models have been developed taking into account a homogeneous time point across patients, such as the onset of symptoms, for the assessment of disease progression.

This study aims at identifying a panel of clinical and laboratory parameters that could support the prediction of disease progression based on symptoms onset in hospitalized patients with COVID-19.

## Methods

### Study design and participants

This is a prospective cohort study. All adult patients (aged ≥ 18 years) with microbiologically confirmed SARS-CoV-2 infection admitted from 1st March to 31st May, 2020 at the University Hospital of Verona were prospectively followed during the hospital stay and included in the COVID 19-VR registry (registered on ClinicalTrials.gov, 18/05/2020, Number: NCT04497194). The study protocol is performed in accordance with the relevant guidelines. The study was approved by the hospital Institutional Review Board (“Comitato etico per la sperimentazione clinica delle province di Verona e Rovigo”, 2577CESC). A written informed consent for study participation was obtained from the patients or from the legal guardian on enrolment. Infection with SARS-CoV-2 was microbiologically defined by a positive real-time reverse-transcriptase polymerase chain reaction (RT-PCR) assay from nasopharyngeal swab.

### Data collection

Patient-level demographic (age, sex, ethnicity), chronic underlying diseases (hypertension and/or coronary artery disease, diabetes, dyslipidaemia, pulmonary disease (chronic obstructive pulmonary disease, active tuberculosis, asthma or obstructive sleep apnoea syndrome), chronic kidney disease, neoplastic disease, thyroid disorders, neurological/psychiatric disorders, liver diseases, obesity (body mass index higher than 30), and clinical parameters (body temperature, respiratory rate, blood pressure, heart rate, peripheral oxygen saturation) were gathered at study inclusion and entered into a pre-defined case report form. Laboratory parameters, including lymphocytes (reference range: 120–400 10^9^/L) and neutrophils (reference range: 180–800 10^9^/L) count, LDH (reference range: 135–214 U/L), CRP (reference range: < 5 mg/L), aspartate aminotransferase (AST, reference range: 5–45 U/L), alanine aminotransferase (ALT, reference range: 5–45 U/L)), creatine kinase (CK, reference range: 30–200 U/L) were extracted from routine blood testing at fixed time points: at hospital admission, day-5 (± 1) and day-10 (± 1) from onset of symptoms and in case of transfer to ICU/sub-intensive unit, discharge, and death.

### Outcomes

COVID-19 progression was defined as PaO_2_/FiO_2_ ratio < 200 and/or transfer to ICU and/or in-hospital death occurring by day-11 after symptoms onset.

### Statistical analysis

Descriptive statistics included means with standard deviations (SD) and frequency analyses (percentages) for categorical variables at day 5 after onset of symptoms. T-test for independent samples, χ^2^ test, or Fisher's test were applied to compare differences between patients with or without disease progression. Logistic regression was performed to evaluate univariate and multivariate associations with the outcome. Results were presented as odds ratio (OR) and 95% confidence interval (95% CI). A *p*-value less than 0.05 was deemed as statistically significant. In order to enter the model as categorical variable, the cut-off of each laboratory parameter was set at the best point of sensitivity and specificity identified through the receiver operating characteristic (ROC) curve. The selected parameters with correspondent cut-offs were: CRP > 80 mg/L, neutrophil lymphocyte ratio (NLR) > 4.5, LDH > 250 U/L, CK > 80 U/L, and ALT > 40 U/L. Candidate predictors to enter the logistic multivariate regression were variables with *p* < 0.05 in univariate analysis. Variables independently associated with the outcome at multivariate analysis together with those deemed as critically relevant in accordance with literature evidence and clinical experience were selected to fit the prediction model. The performance of the model was assessed by calculating the ROC curves and the corresponding area under the curve (AUC) values. The Hosmer–Lemeshow test was applied to assess the goodness-of-fit. All statistical analyses were carried out using International Business Machines (IBM) Statistical Package for the Social Sciences (SPSS) 21 (IBM Corporation: Armonk, NY, 10504).

## Role of the funding source

The funders of the study did not have any role in data collection and in study design, data analysis, data interpretation, or writing of the report. The corresponding author had full access to all data in the study and had final responsibility for the decision to submit for publication.

## Results

A total of 235 patients with COVID-19 were prospectively included in the cohort. Demographic and clinical characteristics of patients enrolled in the study are displayed in Table 1. The majority of patients were male (148, 63%) and the mean age was 71 (SD 15.9). One hundred and ninety patients (81%) suffered from at least one underlying illness, most frequently cardiovascular disease (47%), neurological/psychiatric disorders (35%), and diabetes (21%).

**Table 1:** Demographic and clinical characteristics of patients by COVID-19 disease progression^a^

	TOT (*n* = 235)	Disease progression^a^		Univariate	
		No (*n* = 147)	Yes (*n* = 88)	OR (95% CI)	*p-*value
Age (years) > 65	157 (67%)	90 (61%)	67 (76%)	2.02 (1.12 to 3.65)	0.020
Gender (female)	87 (37%)	58 (40%)	29 (33%)	0.75 (0.43 to 1.31)	0.318
Comorbidities (any)	190 (81%)	119 (81%)	71 (81%)	0.98 (0.50 to 1.92)	0.959
Diabetes	40/190 (21%)	27/119 (23%)	13/71 (18%)	0.76 (0.37 to 1.59)	0.475
Cancer	33/190 (17%)	22/119 (19%)	11/71 (16%)	0.81 (0.37 to 1.79)	0.599
Cardiovascular diseases	90/190 (47%)	45/119 (38%)	45/71 (63%)	2.85 (1.55 to 5.23)	0.001
Renal diseases	39/190 (21%)	20/119 (17%)	19/71 (27%)	0.81 (0.89 to 3.69)	0.103
Respiratory diseases	34/190 (18%)	21/119 (18%)	13/71 (18%)	1.05 (0.49 to 2.25)	0.908
Liver diseases	6/190 (3%)	4/119 (3%)	2/71 (3%)	0.83 (0.15 to 4.67)	0.836
BMI > 30	16/190 (8%)	12/119 (10%)	4/71 (6%)	0.53 (0.17 to 1.72)	0.292
Neurologic/Psychiatric diseases	66/190 (35%)	38/119 (32%)	28/71 (39%)	1.39 (0.75 to 2.56)	0.294
Symptoms					
Fever	193 (82%)	120 (82%)	73 (83%)	1.09 (0.55 to 2.19)	0.798
Cough	106 (45%)	68 (46%)	38 (43%)	0.88 (0.52 to 1.50)	0.646
Dyspnoea	87 (37%)	41 (28%)	46 (52%)	2.83 (1.63 to 4.92)	0.000
Treatment					
Steroids (at least 5 days)	38/183 (21%)	26/124 (21%)	12/59 (20%)	0.96 (0.45 to 2.07)	0.922
Non-invasive ventilation	26 (11%)	17 (12%)	9 (10%)	0.87 (0.37 to 2.05)	0.752
Invasive mechanical ventilation	30 (13%)	2 (1%)	28 (32%)	33.83 (7.81 to 156.53)	0.000
Outcomes (days), mean (SD)					
Time from symptoms onset to admission	3.6 (3.2)	3.2 (2.4)	4.0 (3.9)	0.78 (− 0.08 to 1.63)	0.076
Length hospital stay	13.2 (11.5)	12.4 (11.2)	14.3 (12)	1.82 (− 1.24 to 4.87)	0.243

BMI: body mass index

^a^Disease progression is a composite outcome defined as: death and/or transfer to ICU within day-11 after symptom onset and/or PaO_2_/FiO_2_ ratio < 200 on day 10 after symptom onset. Results are presented as mean (SD) and mean difference (95% CI) or frequencies (%) and OD (95% CI) as appropriate

The majority of patients had fever prior to admission (193, 82%). Cough and dyspnoea were reported in 106 (45%) and 87 (37%) cases, respectively. The mean time from onset of symptoms and admission to hospital was 3.6 days (SD 3.2), while mean length of hospital stay was 13.2 days (SD 11.5). Patients received antiviral therapy according to national and regional recommendations (see Table 1). Steroids (methylprednisolone or dexamethasone), if prescribed, were administered for at least 5 days. In 26 (11%) cases non-invasive ventilation was required, while 30 (13%) patients underwent invasive mechanical ventilation.

**Fig. 1:**
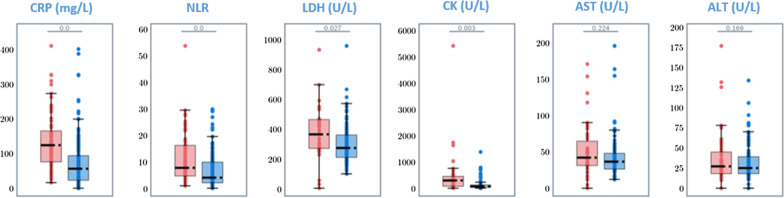
Comparison of laboratory parameters at day-5 from symptoms onset between patents with (red) and without (blue) COVID-19 disease progression. Disease progression is a composite outcome defined as: death and/or transfer to ICU by day-11 after symptom onset, and/or PaO_2_/FiO_2_ ratio < 200 on day 10 after symptom onset. *CRP* C-reactive protein, *NLR* neutrophils to lymphocytes ratio, *LDH* lactate dehydrogenase, *CK* creatine kinase, *AST* aspartate aminotransferase, *ALT* alanine transaminase. *p*-values are reported at the top of each box plot

Eighty-eight (37%) patients experienced poor outcome, e.g. PaO_2_/FiO_2_ ratio < 200 (37 patients, 42%), transfer to ICU (30 patients, 34%), and death (47 patients, 53%) by day-11 after symptoms onset. At univariate analysis (Table 1), patients with poor outcome were more likely to be older than 65 years (OR 2.02; 95% CI 1.12–3.65 years, *p* = 0.020), to suffer from cardiovascular disease (OR 2.85; 95% CI 1.55–5.23, *p* = 0.001), and to have experienced dyspnoea during the 5 days prior to admission (OR 2.83; 95% CI 1.63–4.92, *p* < 0.000). Blood test performed by day-5 of symptoms onset showed that mean values of CRP, NLR, LDH and CK were significantly higher compared with patients not experiencing progression of COVID-19 (Fig. 1). After applying the cut-offs set at the best point of sensitivity and specificity identified through the ROC curve, an association between disease progression and the above mentioned variables showing values above the cut-offs was observed (CRP: OR 7.38; 95% CI 3.83–14.23, *p* < 0.000; NLR: OR 3.43; 95% CI 1.77–6.64, *p* < 0.000; LDH: OR 6.70; 95% CI 2.14–21.04, *p* = 0.001; and CK: OR 3.83; 95% CI 1.56–9.39, *p* = 0.003) (Table 2).

**Table 2:** Univariate analysis of laboratory parameters by COVID-19 progression^a^

	TOT (*n* = 235)	Disease progression^a^		Univariate	
		No (*n* = 147)	Yes (*n* = 88)	OR (95% CI)	*p-*value
Laboratory parameters					
CRP > 80 mg/L	87/196 (44%)	34/124 (27%)	53/72 (74%)	7.38 (3.83 to 14.23)	0.000
NLR > 4.5	104/184 (57%)	54/117 (46%)	50/67 (75%)	3.43 (1.77 to 6.64)	0.000
LDH > 250 U/L	68/104 (65%)	37/69 (54%)	31/35 (89%)	6.70 (2.14 to 21.04)	0.001
CK > 80 U/L	56/102 (55%)	29/66 (44%)	27/36 (75%)	3.83 (1.56 to 9.39)	0.003
ALT > 40 U/L	35/148 (24%)	20/94 (21%)	15/54 (28%)	1.42 (0.66 to 3.09)	0.371
At least 3 abnormal laboratory values^b^	66/210 (31%)	29 (22%)	37 (49%)	3.56 (1.93 to 6.56)	0.000

*CRP* C-reactive protein, *NLR* neutrophils to lymphocytes ratio, *LDH* lactate dehydrogenase, *CK* creatine kinase, *AST* aspartate aminotransferase, *ALT* alanine transaminase

^a^Disease progression is a composite outcome defined as: death and/or transfer to ICU within day-11 after symptom onset, and/or PaO_2_/FiO_2_ ratio < 200 on day 10 after symptom onset

^b^Within: CRP > 80 mg/L; NLR > 4.5; LDH > 250 U/L; CK > 80 U/L; ALT > 40 U/L. Results are presented as mean (SD) and mean difference (95% CI) or frequencies (%) and OD (95% CI), as appropriate

**Fig. 2:**
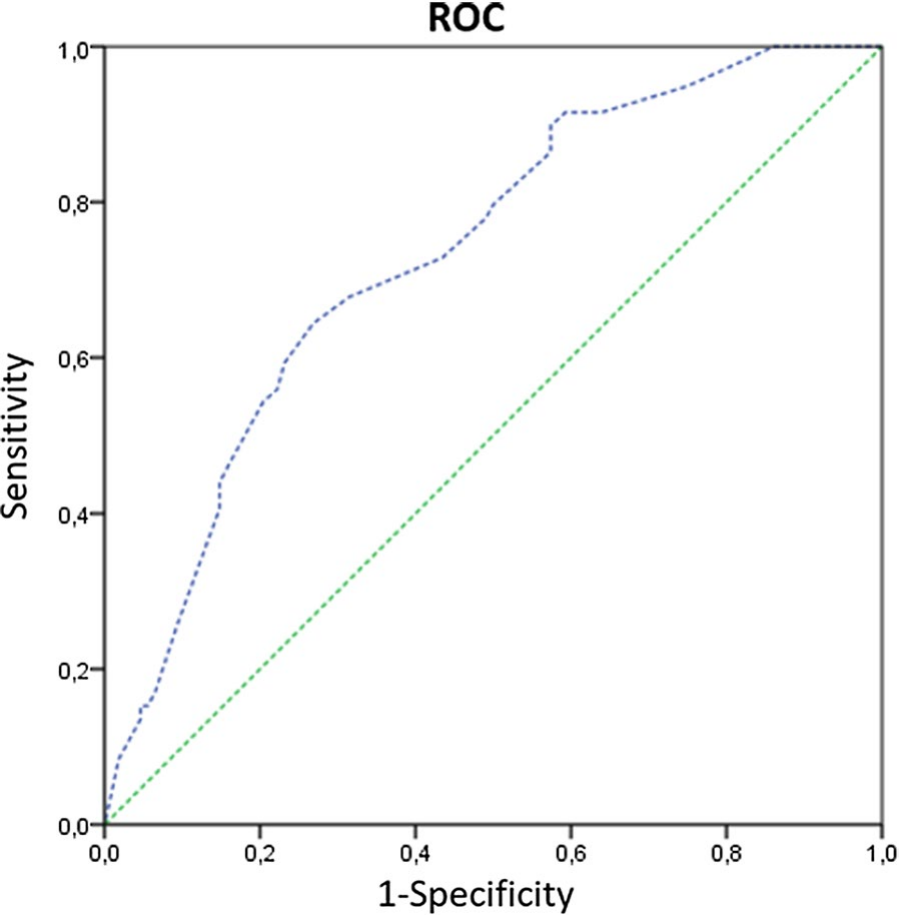
COVID-19 disease progression by day-11 after onset of symptoms: Receiver operator characteristic (ROC) curve for discrimination, Area under the curve (AUC) = 0.733 (95% CI 0.655–0.810). Hosmer–Lemeshow Chi-squared = 8.061, *p* = 0.427; DF = 8). Variables included in the model: age > 65 years, male sex, presence of cardiovascular disease, at least three abnormal parameters at day-5 after symptom onset within: CRP > 80 mg/L; NLR > 4.5; LDH > 250 U/L; CK > 80 U/L; ALT > 40 U/L, and history of dyspnoea prior to admission

Multivariate logistic regression showed that at day 5 presence of cardiovascular disease (OR 2.29; CI 95% 1.12–4.68; *p* = 0.023), dyspnoea (OR 2.40; 95% CI 1.19–4.84*; p* = 0.015) and at least three abnormal laboratory findings among CRP > 80 U/L, ALT > 40 U/L, NLR > 4.5, LDH > 250 U/L, and CK > 80 U/L (OR 2.80; 95% CI 1.35–5.81; *p* = 0.006) were independently associated with COVID-19 progression (Table 3). A model combining the above mentioned variables with age > 65 years old and male sex showed an AUC of 0.73 (95% CI 0.66–0.81) for predicting disease progression. The Hosmer–Lemeshow goodness of fit test did not reach statistical significance (*p* = 0.427), indicating a good match of predicted risk over observed risk. The ROC curve for COVID-19 progression is displayed in Fig. 2.

**Table 3:** Multivariate analysis of clinical characteristics and laboratory parameters by COVID-19 progression

	OR (95% CI)	*p-*value
Age (years) > 65	1.53 (0.63 to 3.67)	0.345
Cardiovascular diseases	2.29 (1.12 to 4.68)	0.023
Dyspnoea	2.40 (1.19 to 4.84)	0.015
At least 3 abnormal laboratory values^a^	2.80 (1.35 to 5.81)	0.006
Antibiotic therapy	1.16 (0.57 to 2.36)	0.691

^a^Within: CRP > 80 mg/L; NLR > 4.5; LDH > 250 U/L; CK > 80 U/L; ALT > 40 U/L

## Discussion

This study proposes an easy-to-use panel of five clinical and laboratory parameters to aid in the prediction of disease progression in COVID-19 patients. The model including age > 65 years, male sex, cardiovascular disease, dyspnoea and at least three abnormal blood parameters among CRP (> 80 U/L), ALT (> 40 U/L), NLR (> 4.5), LDH (> 250 U/L), and CK (> 80 U/L) shows fair discrimination ability (AUC 0.73). To our knowledge, this is the first study assessing predictors of disease progression at specific time-points starting from the onset of COVID-19 symptoms.

Since the start of the pandemic, a wide range of predictive models and scores has been published with the common goal of informing clinical decision making and optimising resource allocation. An observational cohort of 1157 patients acutely admitted to two London hospitals analysed several demographics, clinical, laboratory and imaging factors likely to predict mortality, highlighting a correlation of male sex, older age, hypertension, chronic lung diseases and higher levels of lymphocytes, CRP and creatinine, among the others, with critical care admission and/or death [7]. A systematic review of ten prognostic models revealed that the most reported predictors of disease progression and mortality were age, sex, CRP, LDH and lymphocyte count [9]. Recently, the predictive value of NLR measured at hospital admission has been assessed in a prospective cohort, showing a high value in predicting disease deterioration, shock and death (all the areas under the curve > 0.80) [13]. The vast majority of these models assessed the predictors by considering the hospital admission as baseline time-point [10–13], and share therefore the common drawback of including patients at varying stages of the disease.

In our cohort, more than one-third (37%) of patients experienced disease progression by day-11 of symptoms onset. Descriptive studies on clinical course of COVID-19 revealed that the median time from onset of illness to acute respiratory distress syndrome and to ICU admission was 8–12 days and 9.5–12 days, respectively [14]. These findings would seem to indicate that clinical deterioration with the need of higher level of care may occur at a very early stage of the disease and suggest that setting “onset of symptoms” as baseline time-point leads to inclusion of patients with homogeneous disease length.

Older age and the presence of cardiovascular comorbidities were associated with a higher risk of unfavourable outcome, as highlighted by other studies [7, 15, 16]. Comorbidities, secondary bacterial infections, and altered cellular and humoral immune functions are more common in the elderly, thus increasing the risk of developing severe illness. A meta-analysis of 51 studies including a total of over 48,000 patients with confirmed COVID-19 infection showed that fatal outcomes with COVID-19 infection were strongly associated with diabetes, hypertension and cardiovascular diseases across all age groups, thus suggesting that the risk of poor outcome associated with cardiovascular diseases may be not affected by age [17]. In the present cohort, no statistically significant differences between male and female were observed with regards to the outcome, while in most of the studies the severity and mortality of COVID-19 have been reported to be higher in males than in females. Sex-related differences in immune system responses to pathogens has been observed, with female eliciting higher immune responses. Furthermore, RNA clearance has been reported to be delayed in male patients with COVID-19 [18].

Laboratory assessment on day-5 of symptoms onset showed several parameters with significant mean differences between patients with or without poor outcome. In particular, poor outcome was associated with higher NLR, CRP, LDH, and CK, according with several studies [12, 19]. Lymphopenia and elevated neutrophil count suggest an alteration of lymphocyte function and are associated with elevated secretion of IL-6 and TNF-alpha, since these inflammatory markers contribute to lymphocyte apoptosis as well as decreased proliferation of lymphocytes [20]. Several mechanisms are responsible of this alteration, including direct infection of the lymphocytes with SARS-CoV-2 virus, causing lymphocyte death or dysfunction. Furthermore, the pro-inflammatory state with increased cytokines is inversely correlated with the induction of granulopoiesis (resulting in neutrophilia) and suppressed lymphopoiesis in the bone marrow of patients with SARS‐CoV infection [21].

This inflammatory state, when persistent and uncontrolled, may lead to acute respiratory distress syndrome and rapid deterioration of the clinical conditions. IL-6 upregulates hepatic CRP production, thus suggesting the plausibility of the clinical use of this inflammatory biomarker as a prognosis predictor for COVID-19 patients [9]. Similarly, LDH blood level increases in case of cell damage and is a marker of various inflammatory states. A systematic review and meta-analysis of 28 studies reporting LDH levels in severe vs. non-sever COVID-19 patients confirmed that LDH level can be used as a COVID-19 severity marker and is a predictor of survival [22] CK, a marker of muscle damage, has been associated with a more severe COVID-19. Currently, it remains unclear whether increased levels of CK in COVID-19 patients is due to a virus‐triggered inflammatory response or direct muscle toxicity [23]. Elevation of liver transaminases during SARS-CoV-2 infection has been frequently reported. Possible pathophysiology mechanisms include direct effect of the virus, liver injury mediated by uncontrolled immune response drug toxicity and ischemic hepatitis due to multiorgan dysfunction [24]. Based on these evidences and on our findings, patients affected by COVID-19 may benefit from blood testing including inflammation markers, complete blood count and transaminases within the 1st week after symptoms onset to evaluate the risk of developing a life-threatening disease.

In a recent work, a large group of Italian experts was invited to complete an online survey through the PAPRIKA (Potentially All Pairwise RanKings of all possible Alternatives) method [25] with the aim of determining the weights of several criteria for prioritizing COVID-19 patients for hospitalization [26]. Among a list of criteria, including age, body mass index, comorbidities, findings at chest X-ray, CRP, and duration of symptoms among others, the highest weights were attributed to PaO_2_ and peripheral oxygen saturation, denoting the well-known central role of respiratory findings in the assessment of the risk of rapid deterioration of COVID-19 patients. These findings, alongside those outlined in our study, suggest that in addition to considering symptoms onset and duration, the typology of symptoms should also be taken into account in an early risk assessment of COVID-19 deterioration.

This study has several limitations. First, the sample size limits the accuracy of the findings. Second, the study was performed in a single centre, hampering the generalizability and the applicability of the results to other settings. Third, the model did not undergo external validation and might be at risk of overfitting. However, we tried to mitigate overfitting by decreasing the number of predictors. The results from this study highlight the importance of relying on homogeneous populations with same length of disease in order to build the best possible prediction model for disease progression in COVID-19 patients and allow optimal treatment and resource allocation.

This study shows how an easy-to-use panel of five laboratory and clinical parameters applied in patients with COVID-19 at day-5 of symptoms onset can predict disease progression with fair discriminatory power. It further suggests that the onset of symptoms might represent a useful and reliable baseline time-point for developing prognostic models. The assessment of few variables at the right time may contribute to early identification of patients at major risk of developing life-threatening COVID-19. The model can also play a role as a tool to increase homogeneity of population in clinical trials on COVID-19 treatment in hospitalized patients. A validation study is needed to evaluate whether this model can reliably inform decision making and identify proper level-of-care requirements for hospitalized patients.

## Data Availability

The datasets analysed during the current study are available from the corresponding author on reasonable request.
